# The use of weighted multiple linear regression to estimate QTL × QTL × QTL interaction effects of winter wheat (*Triticum aestivum* L.) doubled-haploid lines

**DOI:** 10.1007/s13353-023-00795-3

**Published:** 2023-10-25

**Authors:** Adrian Cyplik, Dominika Piaskowska, Paweł Czembor, Jan Bocianowski

**Affiliations:** 1 Department of Mathematical and Statistical Methods, Poznań University of Life Sciences, Wojska Polskiego 28, 60-637 Poznań, Poland; 2 Plant Breeding and Acclimatization Institute – National Research Institute, Department of Applied Biology, Radzików 05-870 Błonie, Poland

**Keywords:** Three-way epistasis, Weighted regression, Doubled haploid lines, Resistance, *Triticum aestivum*

## Abstract

Knowledge of the magnitude of gene effects and their interactions, their nature, and contribution to determining quantitative traits is very important in conducting an effective breeding program. In traditional breeding, information on the parameter related to additive gene effect and additive-additive interaction (epistasis) and higher-order additive interactions would be useful. Although commonly overlooked in studies, higher-order interactions have a significant impact on phenotypic traits. Failure to account for the effect of triplet interactions in quantitative genetics can significantly underestimate additive QTL effects. Understanding the genetic architecture of quantitative traits is a major challenge in the post-genomic era, especially for quantitative trait locus (QTL) effects, QTL–QTL interactions, and QTL–QTL–QTL interactions. This paper proposes using weighted multiple linear regression to estimate the effects of triple interaction (additive–additive–additive) quantitative trait loci (QTL–QTL–QTL). The material for the study consisted of 126 doubled haploid lines of winter wheat (Mandub × Begra cross). The lines were analyzed for 18 traits, including percentage of necrosis leaf area, percentage of leaf area covered by pycnidia, heading data, and height. The number of genes (the number of effective factors) was lower than the number of QTLs for nine traits, higher for four traits and equal for five traits. The number of triples for unweighted regression ranged from 0 to 9, while for weighted regression, it ranged from 0 to 13. The total *aaa*_*gu*_ effect ranged from − 14.74 to 15.61, while *aaa*_*gw*_ ranged from − 23.39 to 21.65. The number of detected threes using weighted regression was higher for two traits and lower for four traits. Forty-nine statistically significant threes of the additive-by-additive-by-additive interaction effects were observed. The QTL most frequently occurring in threes was 4407404 (9 times). The use of weighted regression improved (in absolute value) the assessment of QTL–QTL–QTL interaction effects compared to the assessment based on unweighted regression. The coefficients of determination for the weighted regression model were higher, ranging from 0.8 to 15.5%, than for the unweighted regression. Based on the results, it can be concluded that the QTL–QTL–QTL triple interaction had a significant effect on the expression of quantitative traits. The use of weighted multiple linear regression proved to be a useful statistical tool for estimating additive-additive-additive (*aaa*) interaction effects. The weighted regression also provided results closer to phenotypic evaluations than estimator values obtained using unweighted regression, which is closer to the true values.

## Introduction

Wheat (*Triticum aestivum* L.) is a crucial staple crop with significant economic importance. It contributes substantially to the daily calorie intake of Europeans and people worldwide (Erenstein et al. 2022). Wheat production worldwide has seen significant growth over the years, driven by improved technology, adoption of high-yielding and disease-resistant varieties, better management practices, and supportive policies and institutions (Tadesse et al. 2019). One of the major factors limiting the achievement of consistent and stable growth in wheat yield increase is disease, including Septoria tritici blotch (STB) caused by the fungus *Zymoseptoria tritici* (teleomorph *Mycosphaerella graminicola*) (Figueroa et al. 2018; Tabib Ghaffary et al. 2018). The disease occurs in most wheat-growing areas around the world and is characterized by high diversity and complex population structure in local *Z. tritici* populations. This is primarily due to high gene flow within and between populations and frequent sexual reproduction (Figueroa et al. 2018; Orton et al. 2011).

Resistance to *Z. tritici* in wheat can exhibit both qualitative and quantitative nature, as described by Brown et al. (2015). Qualitative resistance is usually isolate-specific and nearly complete. It is regulated by major genes that follow the gene–for–gene concept, as shown in studies such as Brading et al. (2002), and includes at least 22 major genes associated with *Z. tritici* resistance in wheat (Tabib Ghaffary et al. 2018; Brown et al. 2015; Yang et al. 2018). On the other hand, resistance to STB can also be quantitative, usually lacking isolate specificity and involving polygenic inheritance. In addition to the major resistance loci, nearly 100 regions of the genome carrying quantitative trait loci (QTLs) and meta–QTLs with small effects have been identified (Brown et al. 2015; Piaskowska et al. 2021; Langlands-Perry et al. 2022). Significant loci associated with STB resistance have also been identified by genome-wide association studies (GWAS) (Kollers et al. 2013; Miedaner et al. 2013; Gurung et al. 2014; Odilbekov et al. 2019; Louriki et al. 2021; Yang et al. 2022). This method uses a diverse panel of genotypes consisting of varieties with a broad spectrum of resistance responses and diverse genetic backgrounds, thus bypassing the time-consuming process of crossing and crossing and breeding mapping progeny.

The idea of genetic interactions has been known for more than a century, since the work of Bateson and Mendel (1902). Since multiple genetic loci often influence complex traits, several statistical methods have been developed to identify epistatic effects (Yi et al. 2007). Most studies focus on analyzing the association between individual genes and phenotypic traits (Bocianowski and Krajewski 2009; Tura et al. 2020; Khan et al. 2021; Ma et al. 2022; Devi et al. 2019; Yang et al. 2021). Although pairwise interactions are widely used, higher-order interactions are often overlooked. The requirement for complete and precise data, which has been a challenge to obtain until recently, has been a limiting factor for progress in this type of study. However, the knowledge about higher-order interactions may be missing in understanding the mechanics of heritability and the relationship between genotype and phenotype. While there is still much to learn, recognizing and studying these complex interactions can provide a complete understanding of genetic inheritance (Cyplik et al. 2023; Cyplik and Bocianowski 2023).

Genetic interactions refer to the way genes and their products determine a phenotype. These interactions can range from simple additive effects to more complex epistatic or pleiotropic products. Epistasis occurs when the expression of one gene depends on the presence or absence of another gene, while pleiotropy refers to a single gene affecting multiple traits (Li et al. 2011; Krajewski et al. 2012; Ku et al. 2012; Beheshtizadeh et al. 2018; Dhariwal et al. 2018; Smeda et al. 2018; Ali et al. 2022; Pundir et al. 2022; Yusuf et al. 2022). Therefore, understanding genetic interactions is crucial for advancing our knowledge of genetics and developing effective selection methods (Wang et al. 2012; Ali et al. 2020; Cullis et al. 2020; Labroo et al. 2021; Raffo et al. 2022).

QTL by QTL by QTL interactions refer to a complex interaction between multiple genetic loci that affect a quantitative trait (Mackay 2014). In these interactions, the effect of one QTL on a trait depends on the presence or absence of other QTLs, resulting in a complex genetic network that determines the phenotype (Jarvis and Cheverud 2011; Li et al. 2016; 2019). QTL by QTL by QTL interactions are crucial for understanding complex relationships in which multiple genetic and environmental factors determine phenotype (Cyplik et al. 2023). Analysis of these interactions requires advanced statistical and computational methods that can handle the complexity of the genetic network involved (Yi et al. 2007). Traditional methods of measuring genetic interactions, such as pairwise analysis, are insufficient to capture the complexity of these interactions (Hartman et al. 2001; Brem and Kruglyak 2005). Newer techniques, such as machine learning and network analysis, are being developed to address this problem.

In addition, experimental design is critical for measuring QTL by QTL by QTL interactions, and careful consideration of sample size, statistical power, and environmental factors are required to ensure the accuracy of the results. Despite the challenges involved, understanding QTL by QTL by QTL interactions is critical to improving our understanding of the genetic basis of complex traits. Genetic interactions play a critical role in agriculture, where they are used to improve crop yields, enhance resistance to pests and disease, and increase crop nutrient content (Farokhzadeh et al. 2019; Barmukh et al. 2021; Arif et al. 2022; Bokore et al. 2022).

Understanding the complex interactions between genes that control these traits is essential for developing new crop varieties as part of plant breeding programs. For example, epistatic interactions between multiple genes can contribute to the expression of desirable traits, such as drought tolerance or increased yield. Identifying and exploiting these interactions can significantly improve crop productivity (Singhal et al. 2022).

In addition, genetic interactions can be used to develop crops with improved nutrient content, such as increased levels of vitamins and minerals. Advances in molecular genetics and genomics have provided new tools and approaches for analyzing genetic interactions, enabling more precise and efficient plant breeding. As such, genetic interactions are a critical component of modern agriculture and will continue to play an important role in addressing global food security challenges in the future (Bonas and Van der Ackerveken 1999; Graham et al. 2001; Taylor and Ehrenreich 2015).

The reason for the difficulty in measuring triple gene interactions is simple. Using phenotypic data, it is only possible to estimate the overall effect of all hypothetical triple gene interactions contributing to a particular trait. However, using marker data, which can be more precisely mapped in the genome, makes it possible to estimate individual effects of gene-by-gene-by-gene interactions, while limiting the number of QTL-by-QTL-by-QTL interactions for practical reasons. The sum of these individual effects is usually smaller than the phenotypic estimate, which can be further complicated by the lack of markers in the regions where the genes are located (Cyplik and Bocianowski 2023).

In addition to the previously discussed factors, other potential reasons for differences in estimated values should be considered. The previously mentioned values refer to QTL-by-QTL-by-QTL interaction effects, which were calculated using a basic method: multiple linear regression on marker data. However, this paragraph suggests that modifying the regression by incorporating empirical weights improves the agreement between phenotype- and genotype-based estimates (Cyplik et al. 2023). The study described in the paper indicates that these modifications can help account for additional sources of variation in the data.

The purpose of this study was to use weighted multiple linear regression to estimate the additive by additive by additive (*aaa*) interaction effects. To compare the estimates of *aaa* obtained by unweighted and weighted methods, phytopathological tests were used, resulting in 18 data sets that included the percentage of leaf area covered by necrosis and pycnidia, as well as the heading dates and height data from the polytunnel tests were used.

## Materials and methods

### Plant material

The data used in this study was produced and published previously by Piaskowska et al. (2021). The purpose of this study was to map STB resistance in the winter wheat cultivar Mandub. A set of 126 doubled-haploid (DH) lines derived from a cross between Mandub (the German cultivar revealed a high resistance level at the seedling and adult plant stages) and the susceptible cultivar Begra was used as the mapping population. Tests were conducted at the seedling (second leaves fully emerged) and adult (flag leaves fully emerged) plant growth stages. Plants were inoculated by spraying with an aqueous suspension of pycnidiospore of one of three selected *Z. tritici* isolates. Evaluation of disease development took place when the necrotic area of the second/flag leaves in the susceptible control variety (Begra) reached approximately 80%. To determine the percentage of leaf area covered by necrosis, leaves were mounted on a self-adhesive foil and photographed. Next, on the same leaves, the area bearing pycnidia was marked with a red and photographed again. To accurately measure disease parameters, images were analyzed using WinCam software (Regent Instruments, Inc. 2004). A total of six tests were conducted, one for each of the selected *Z. tritici* isolates at both growth stages, resulting in 18 sets of phenotypic data that included necrotic area, pycnidia bearing area, and heading date and plant height for adult plant experiments (Table 1). Genotypic data were obtained using the DArTseq platform (Diversity Arrays Technology, Pty. Ltd., Australia). The linkage map provided by DArT P/L consisted of 5899 molecular markers. Markers were assigned to 25 linkage groups, resulting in a map with a total length of 2666 cM. Missing marker values were estimated based on missing flanking marker data (Martinez and Curnow 1994).

**Table 1:** List of traits of resistance to *Septoria tritici* blotch analyzed for Mandub × Bagra doubled haploid lines of winter wheat as well as minimal and maximal values of average for lines, means for all lines, phenotypic estimates of the total additive-by-additive-by-additive effect (aaap) and the number of genes (the number of effective factors)

Trait number	Trait	Isolate	Experimental condition	Min. effect	Max. effect	Lines mean	*aaa*_p_	The number of genes (the number of effective factors)
1	Percentage of necrosis leaf area	IPO86036	Polytunnel	6.67	96.82	51.26	0.48	4
2	Percentage of leaf area covered by pycnidia			0.26	58.57	27.36	2.06*	3
3	Percentage of necrosis leaf area		Plant growth chamber	2.65	98.26	45.48	4.97**	2
4	Percentage of leaf area covered by pycnidia			0.11	63.47	18.54	13.25***	3
5	Percentage of necrosis leaf area	IPO92006	Polytunnel	1.71	94.58	51.57	–3.42*	3
6	Percentage of leaf area covered by pycnidia			0	47.31	13.28	10.37***	3
7	Percentage of necrosis leaf area		Plant growth chamber	7.33	72.55	35.76	4.18**	3
8	Percentage of leaf area covered by pycnidia			0.39	36.46	13.20	5.23***	4
9	Percentage of necrosis leaf area	IPO88004	Polytunnel	7.12	99.8	80.30	–26.84***	3
10	Percentage of leaf area covered by pycnidia			0.01	59.65	13.87	15.96***	3
11	Percentage of necrosis leaf area		Plant growth chamber	5.73	99.09	59.98	–7.57**	2
12	Percentage of leaf area covered by pycnidia			1.13	88.57	39.78	5.07**	2
13	Heading data	IPO86036	Polytunnel	52.5	95	73.27	0.48	5
14		IPO92006		52.5	100	79.15	–2.90	5
15		IPO88004		50	105	82.84	–5.34*	4
16	Height	IPO86036	Polytunnel	150	159	153.07	1.43	5
17		IPO92006		150	159	153.01	1.49	4
18		IPO88004		146	160.5	152.35	0.90	5

* *p* < 0.05; ** *p* < 0.01; *** *p* < 0.001

### Statistical analysis

Assuming that *n* homozygous (doubled haploid, DH; recombinant inbred, RI) plant lines were observed in the experiment, the following was obtained: *n*-vector of phenotypic mean observations $${\varvec{y}}=\left[\begin{array}{cc}\begin{array}{cc}{y}_{1}& {y}_{2}\end{array}& \begin{array}{cc}\cdots & {y}_{n}\end{array}\end{array}\right]{\prime}$$ and *q n*-vectors of marker genotype observations **m**_l_, *l* = 1, 2, …, q. The *i*th element (*i* = 1, 2, …, *n*) of **m**_l_-vector is equal to − 1 or 1, depending on the parent's genotype exhibited by the *i*th line.

### Estimation based on the phenotype

The total additive × additive × additive interaction of homozygous loci (three-way epistasis) effect based on phenotypic (${aaa}_{p}$) observations ***y*** can be estimated by the formula (Cyplik and Bocianowski 2023):

1 $$\widehat{{aaa}_{p}}=\frac{1}{2}\left({L}_{max}+{L}_{min}\right)-\overline{L }$$

where ${L}_{min}$ and ${L}_{max}$ are the lines with minimal and maximal mean value, respectively; $\overline{L }$ is the mean of all inbred lines. The test statistic to verify the hypothesis about ${aaa}_{P}$ different than zero is given by (Cyplik et al. 2022):

2 $${F}_{{aaa}_{p}}=\frac{{MS}_{{aaa}_{p}}}{{MS}_{e}}$$

where ${MS}_{{aaa}_{p}}$ and ${MS}_{e}$ are mean squares for epistasis ${aaa}_{p}$ and residual, respectively. The number of genes (number of effective factors) obtained based on phenotypic observations only was calculated using a formula presented by Kaczmarek et al. (1988).

### Estimation based on the genotype

The *aaa* was estimated under the presumption that the observed molecular markers accurately identified the genes accountable for the characteristic. The variability of the characteristic and model observations for the lines can be established by selecting from all observed markers *p* as:

3 $${\varvec{y}}=1\mu +{\varvec{X}}{\varvec{\beta}}+{\varvec{Z}}{\varvec{\gamma}}+{\varvec{W}}{\varvec{\delta}}+{\varvec{e}}$$

where **1**, the *n*-vector of ones; $\mu$, overall mean; **X**, *(n* × *p)*-matrix of the form $${\varvec{X}}=\left[\begin{array}{cc}\begin{array}{cc}{{\varvec{m}}}_{{l}_{1}}& {{\varvec{m}}}_{{l}_{2}}\end{array}& \begin{array}{cc}\cdots & {{\varvec{m}}}_{{l}_{p}}\end{array}\end{array}\right]$$, *l*_*1*_, *l*_*2*_, …, *l*_*p*_ ∈ {1, 2, …, *q*}; ${\varvec{\beta}}$, the *p*-vector of unknown parameters of the form $${\varvec{\beta}}\boldsymbol{^{\prime}}=\left[\begin{array}{cc}\begin{array}{cc}{a}_{{l}_{1}}& {a}_{{l}_{2}}\end{array}& \begin{array}{cc}\cdots & {a}_{{l}_{p}}\end{array}\end{array}\right]$$; ***Z***, matrix whose columns are products of some columns of matrix ***X***; ${\varvec{\gamma}}$, the vector of unknown parameters of the form $${\varvec{\gamma}}\boldsymbol{^{\prime}}=\left[\begin{array}{cc}\begin{array}{cc}{aa}_{{l}_{1}{l}_{2}}& {aa}_{{{l}_{1}l}_{3}}\end{array}& \begin{array}{cc}\cdots & {aa}_{{{l}_{p-1}l}_{p}}\end{array}\end{array}\right]$$; ***W***, matrix whose columns are three-way products of some columns of matrix ***X***; ${\varvec{\delta}}$, the vector of unknown parameters of the form $${\varvec{\delta}}\boldsymbol{^{\prime}}=\left[\begin{array}{cc}\begin{array}{cc}{aaa}_{{l}_{1}{l}_{2}{l}_{3}}& {aaa}_{{{l}_{1}{l}_{2}l}_{4}}\end{array}& \begin{array}{cc}\cdots & {aaa}_{{{{l}_{p-2}l}_{p-1}l}_{p}}\end{array}\end{array}\right]$$; ***e***, the *n*-vector of random variables such that *E(e*_*i*_*)* = 0, *Cov(e*_*i*_*, e*_*j*_*)* = 0 for *i* ≠ *j*, *i*, *j* = 1, 2, …, *n*. Parameters ${a}_{{l}_{1}}$, ${a}_{{l}_{2}}$, …, and ${a}_{{l}_{p}}$ are additive effects of genes controlling the trait, parameters ${aa}_{{l}_{1}{l}_{2}}$, ${aa}_{{l}_{1}{l}_{3}}$, …, and ${aa}_{{l}_{p-1}{l}_{p}}$ are additive × additive interaction effects, and parameters ${aaa}_{{l}_{1}{l}_{2}{l}_{3}}$, ${aaa}_{{l}_{1}{l}_{2}{l}_{4}}$, …, and ${aaa}_{{{l}_{p-2}l}_{p-1}{l}_{p}}$ are additive × additive × additive interaction effects. Epistatic and three-way interaction effects were assumed to be shown only by loci with significant additive effects of genes. This assumption significantly reduces the number of potentially significant effects and increases the usefulness of the regression model.

#### Unweighted regression

Denoting by $$\boldsymbol{\alpha }\boldsymbol{^{\prime}}=\left[\begin{array}{cc}\begin{array}{cc}\mu &{\boldsymbol{\beta}}\boldsymbol{^{\prime}}\end{array}& \begin{array}{cc}{\boldsymbol{\gamma}}\boldsymbol{^{\prime}}&{\boldsymbol{\delta}}\boldsymbol{^{\prime}}\end{array}\end{array}\right]$$ and $${\varvec{G}}=\left[\begin{array}{cc}\begin{array}{cc}1& {\varvec{X}}\end{array}& \begin{array}{cc}{\varvec{Z}}& {\varvec{W}}\end{array}\end{array}\right]$$, we obtain the model:

4 $${\varvec{y}}={\varvec{G}}\boldsymbol{\alpha }+{\varvec{e}}$$

If **G** is of full rank, the estimate of ${\boldsymbol{\alpha }}_{{\varvec{u}}}$ from traditional (unweighted) multiple linear regression model is given by (Searle 1982):

5 $$\widehat{{\boldsymbol{\alpha }}_{{\varvec{u}}}}={\left({\varvec{G}}^{\prime}{\varvec{G}}\right)}^{-1}{\varvec{G}}^{\prime}{\varvec{y}}$$

The total three-way epistasis *aaa*_*gu*_ effect of genes influencing the trait from traditional (unweighted) multiple linear regression model can be found as (Cyplik and Bocianowski 2022):

6 $${\widehat{aaa}}_{gu}=\sum\nolimits_{k=1}^{p-2}\sum\nolimits_{\begin{array}{c}{{k}{^{\prime}=2}}\\ {k}^{\prime}\ne k\end{array}}^{p-1}\sum\nolimits_{\begin{array}{c}{{k}^{{\prime}{\prime}}=3}\\ {k}^{{\prime}{\prime}}\ne k^{\prime}\end{array}}^{p}{\widehat{aaa}}_{{l}_{k}{l}_{k^{\prime}}{l}_{k^{{\prime}{\prime}}}}$$

To select markers for model (3), we used the stepwise feature selection method using Akaike information criteria (AIC) (Akaike 1998). This process involved two steps: we initially divided the markers into groups based on the linkage groups they belonged to and applied stepwise feature selection based on AIC. We then combined the remaining markers into one group and repeated the same selection process. The final set of markers remained after combining all the remaining markers into the last group and performing the final feature selection on the model with an additive × additive × additive interaction effect. In the first and second steps, we used a critical significance level of 0.001 resulting from a Bonferroni correction (Province 2001).

#### Weighted regression

A modified version of the trait regression on marker data in this paper is considered by adopting a weighted multiple linear regression, that is, a regression with a diagonal matrix ***W*** of unknown observation variances, which, however, can be empirically found by estimation. In this model the estimate of ${\boldsymbol{\alpha }}_{{\varvec{w}}}$ is:

7 $$\widehat{{\boldsymbol{\alpha }}_{{\varvec{w}}}}={\left({\varvec{G}}^{\prime}{{\varvec{W}}}^{-1}{\varvec{G}}\right)}^{-1}{\varvec{G}}^{\prime}{{\varvec{W}}}^{-1}{\varvec{y}}$$

where ${\varvec{W}}=\left({w}_{ii}\right)$ with ${w}_{ii}$ being the estimated variance for *i*th line, *i* = 1, 2, …, *n*. The selection of markers for the weighted regression is made by the same method as described for the unweighted case.

The total three-way epistasis *aaa*_gw_ effect of genes influencing the trait from the weighted multiple linear regression model can be found as

8 $${\widehat{aaa}}_{gw}=\sum\nolimits_{k=1}^{p-2}\sum\nolimits_{\begin{array}{c}{{k}{^{\prime}=2}}\\ {k}^{\prime}\ne k\end{array}}^{p-1}\sum\nolimits_{\begin{array}{c}{{k}^{{\prime}{\prime}}=3}\\ {k}^{{\prime}{\prime}}\ne k^{\prime}\end{array}}^{p}{\widehat{aaa}}_{{l}_{k}{l}_{k^{\prime}}{l}_{k^{{\prime}{\prime}}}}$$

The coefficients of determination were used to measure how both models (unweighted and weighted) fitted the data and, in this study, were the amount of the phenotypic variance explained by total threes of interactive models.

## Results

The results of the total additive × additive × additive interaction effect estimates obtained are shown in Tables 1, 2, 3, and 4. Tables 1 and 2 contain phenotypic and genotypic analysis for 126 doubled haploid lines of winter wheat (cross Mandub × Begra), respectively. Tables 3 and 4 include genotypic estimates of additive × additive × additive interaction effects for individual QTL × QTL × QTL threes for the same data as above, based on unweighted (*aaa*_*gu*_) and weighted (*aaa*_*gw*_) multiple linear regression and the percentage of variance accounted for, respectively.

**Table 2:** Genotypic estimates of the total additive-by-additive-by-additive effects estimated on the basis of unweighted (*aaa*_*gu*_) and weighted (*aaa*_*gw*_) multiple linear regression

Trait number	QTLs number	Unweighted				Weighted			
		Number of *aaa*_*gu*_	*aaa*_*gu*_ effects			Number of *aaa*_*gw*_	*aaa*_*gw*_ effects		
			Min	Max	Total		Min	Max	Total
1	4	0				0			
2	4	0				0			
3	3	1	15.61	15.61	15.61	1	21.65	21.65	21.65
4	5	9	− 9.15	9.9	− 14.74	8	− 12.72	10.47	− 23.39
5	2	0				0			
6	4	0				0			
7	5	4	− 7.93	6.59	− 13.03	0			
8	3	1	2.686	2.686	2.686	0			
9	1	0				0			
10	3	0				0			
11	4	1	6.8	6.8	6.8	1	11.33	11.33	11.33
12	2	0				0			
13	10	6	− 3.351	3.08	− 0.292	10	− 4.243	4.7	17.441
14	7	2	− 3.113	3.411	0.298	1	− 3.78	− 3.78	− 3.78
15	7	0				4	− 4.73	4.3	− 0.55
16	5	0				0			
17	4	0				0			
18	2	0				0

**Table 3:** Genotypic estimates of the additive × additive × additive interaction effects for the particular QTL × QTL × QTL threes for the 126 doubled haploid lines obtained from the cross Mandub × Begra on the basis of unweighted (*aaa*_*gu*_) and weighted (*aaa*_*gw*_) multiple linear regression

Trait			3		4		7	8	11		13		14		15
QTL1	QTL2	QTL3	*aaa*_*gu*_	*aaa*_*gw*_	*aaa*_*gu*_	*aaa*_*gw*_	*aaa*_*gu*_	*aaa*_*gu*_	*aaa*_*gu*_	*aaa*_*gw*_	*aaa*_*gu*_	*aaa*_*gw*_	*aaa*_*gu*_	*aaa*_*gw*_	*aaa*_*gw*_
1034868	3021909	1678083|F|0–32:T > C-32:T > C							6.8	11.33					
1250712	3945004	1001817	15.61	21.65											
3021909	1226085	1110543|F|0–16:C > T-16:C > T				4.29									
3021909	1226085	2257522|F|0–45:C > T-45:C > T			− 7.11										
3021909	1226085	1678083|F|0–32:T > C-32:T > C			− 4.66	− 7.26									
3021909	1110543|F|0–16:C > T-16:C > T	2257522|F|0–45:C > T-45:C > T			9.9										
3021909	1110543|F|0–16:C > T-16:C > T	1678083|F|0–32:T > C-32:T > C			5.27	8.72									
3021909	2257522|F|0–45:C > T-45:C > T	1678083|F|0–32:T > C-32:T > C			− 9.15	− 12.72									
1226085	1110543|F|0–16:C > T-16:C > T	2257522|F|0–45:C > T-45:C > T			− 5.15	− 9.23									
1226085	1110543|F|0–16:C > T-16:C > T	1678083|F|0–32:T > C-32:T > C			− 6.01	− 8.66									
1226085	2257522|F|0–45:C > T-45:C > T	1678083|F|0–32:T > C-32:T > C			7.35	10.47									
1110543|F|0–16:C > T-16:C > T	2257522|F|0–45:C > T-45:C > T	1678083|F|0–32:T > C-32:T > C			− 5.18	− 9									
1304133	3028296	3944270												− 3.78	
2293689	3935712	4407404										− 3.5			
2293689	1021903|F|0–14:G > A-14:G > A	4407404									− 2.838				
2293689	1130002	1230706										3.596			
2293689	1130002	992306|F|0–7:A > G-7:A > G										4.391			
1130002	1100932	3064357|F|0–7:G > C-7:G > C										− 3.905			
5411566	1042631|F|0–26:G > C-26:G > C	2301499						2.686							
5411566	2301499	3935244					− 6.7								
5411566	4989121|F|0–36:C > T-36:C > T	3935244					− 7.93								
5411566	4989121|F|0–36:C > T-36:C > T	2301499					− 4.99								
3935244	2301499	4989121|F|0–36:C > T-36:C > T					6.59								
1100932	4990085	4992872											3.411		
1100932	3935712	1021903|F|0–14:G > A-14:G > A									− 2.799				
4990085	3028296	2277299													3.89
1230706	1021903|F|0–14:G > A-14:G > A	4407404									3.039	4.489			
1260909	992306|F|0–7:A > G-7:A > G	3064357|F|0–7:G > C-7:G > C										− 4.243			
992306|F|0–7:A > G-7:A > G	3935712	4407404										3.376			
992306|F|0–7:A > G-7:A > G	1021903|F|0–14:G > A-14:G > A	4407404										4.7			
992306|F|0–7:A > G-7:A > G	4407404	3028296										4.336			
3935712	1021903|F|0–14:G > A-14:G > A	4407404									3.08	4.201			
4992872	3028296	992306|F|0–7:A > G-7:A > G													− 4.01
4992872	3028296	2277299													− 4.73
4407404	1021903|F|0–14:G > A-14:G > A	1100932									− 3.351				
4407404	1021903|F|0–14:G > A-14:G > A	992306|F|0–7:A > G-7:A > G									2.577				
3028296	1100932	4990085											− 3.113		
3028296	1860348	2277299													4.3

**Table 4:** Percentage variance accounted for the particular QTL × QTL × QTL threes on the basis of unweighted (*aaa*_*gu*_) and weighted (*aaa*_*gw*_) multiple linear regression

Trait			3		4		7	8	11		13		14		15
QTL1	QTL2	QTL3	*aaa*_*gu*_	*aaa*_*gw*_	*aaa*_*gu*_	*aaa*_*gw*_	*aaa*_*gu*_	*aaa*_*gu*_	*aaa*_*gu*_	*aaa*_*gw*_	*aaa*_*gu*_	*aaa*_*gw*_	*aaa*_*gu*_	*aaa*_*gw*_	*aaa*_*gw*_
1034868	3021909	1678083|F|0–32:T > C-32:T > C							11.3	15.9					
1250712	3945004	1001817	50.0	50.8											
3021909	1226085	1110543|F|0–16:C > T-16:C > T				9.6									
3021909	1226085	2257522|F|0–45:C > T-45:C > T			15.6										
3021909	1226085	1678083|F|0–32:T > C-32:T > C			11.8	15.7									
3021909	1110543|F|0–16:C > T-16:C > T	2257522|F|0–45:C > T-45:C > T			31.1										
3021909	1110543|F|0–16:C > T-16:C > T	1678083|F|0–32:T > C-32:T > C			12.7	23.8									
3021909	2257522|F|0–45:C > T-45:C > T	1678083|F|0–32:T > C-32:T > C			31.2	51.8									
1226085	1110543|F|0–16:C > T-16:C > T	2257522|F|0–45:C > T-45:C > T			11.5	27.0									
1226085	1110543|F|0–16:C > T-16:C > T	1678083|F|0–32:T > C-32:T > C			17.9	23.4									
1226085	2257522|F|0–45:C > T-45:C > T	1678083|F|0–32:T > C-32:T > C			28.7	33.1									
1110543|F|0–16:C > T-16:C > T	2257522|F|0–45:C > T-45:C > T	1678083|F|0–32:T > C-32:T > C			12.4	25.5									
1304133	3028296	3944270												12.6	
2293689	3935712	4407404										11.8			
2293689	1021903|F|0–14:G > A-14:G > A	4407404									8.9				
2293689	1130002	1230706										14.1			
2293689	1130002	992306|F|0–7:A > G-7:A > G										21.6			
1130002	1100932	3064357|F|0–7:G > C-7:G > C										16.7			
5411566	1042631|F|0–26:G > C-26:G > C	2301499						8.8							
5411566	2301499	3935244					16.1								
5411566	4989121|F|0–36:C > T-36:C > T	3935244					22.4								
5411566	4989121|F|0–36:C > T-36:C > T	2301499					8.5								
3935244	2301499	4989121|F|0–36:C > T-36:C > T					15.5								
1100932	4990085	4992872											10.7		
1100932	3935712	1021903|F|0–14:G > A-14:G > A									9.7				
4990085	3028296	2277299													13.6
1230706	1021903|F|0–14:G > A-14:G > A	4407404									11.5	21.7			
1260909	992306|F|0–7:A > G-7:A > G	3064357|F|0–7:G > C-7:G > C										20.2			
992306|F|0–7:A > G-7:A > G	3935712	4407404										12.3			
992306|F|0–7:A > G-7:A > G	1021903|F|0–14:G > A-14:G > A	4407404										25.0			
992306|F|0–7:A > G-7:A > G	4407404	3028296										20.5			
3935712	1021903|F|0–14:G > A-14:G > A	4407404									11.9	19.7			
4992872	3028296	992306|F|0–7:A > G-7:A > G													14.8
4992872	3028296	2277299													20.3
4407404	1021903|F|0–14:G > A-14:G > A	1100932									14.0				
4407404	1021903|F|0–14:G > A-14:G > A	992306|F|0–7:A > G-7:A > G									8.0				
3028296	1100932	4990085											8.9		
3028296	1860348	2277299													15.9

**Fig.1:**
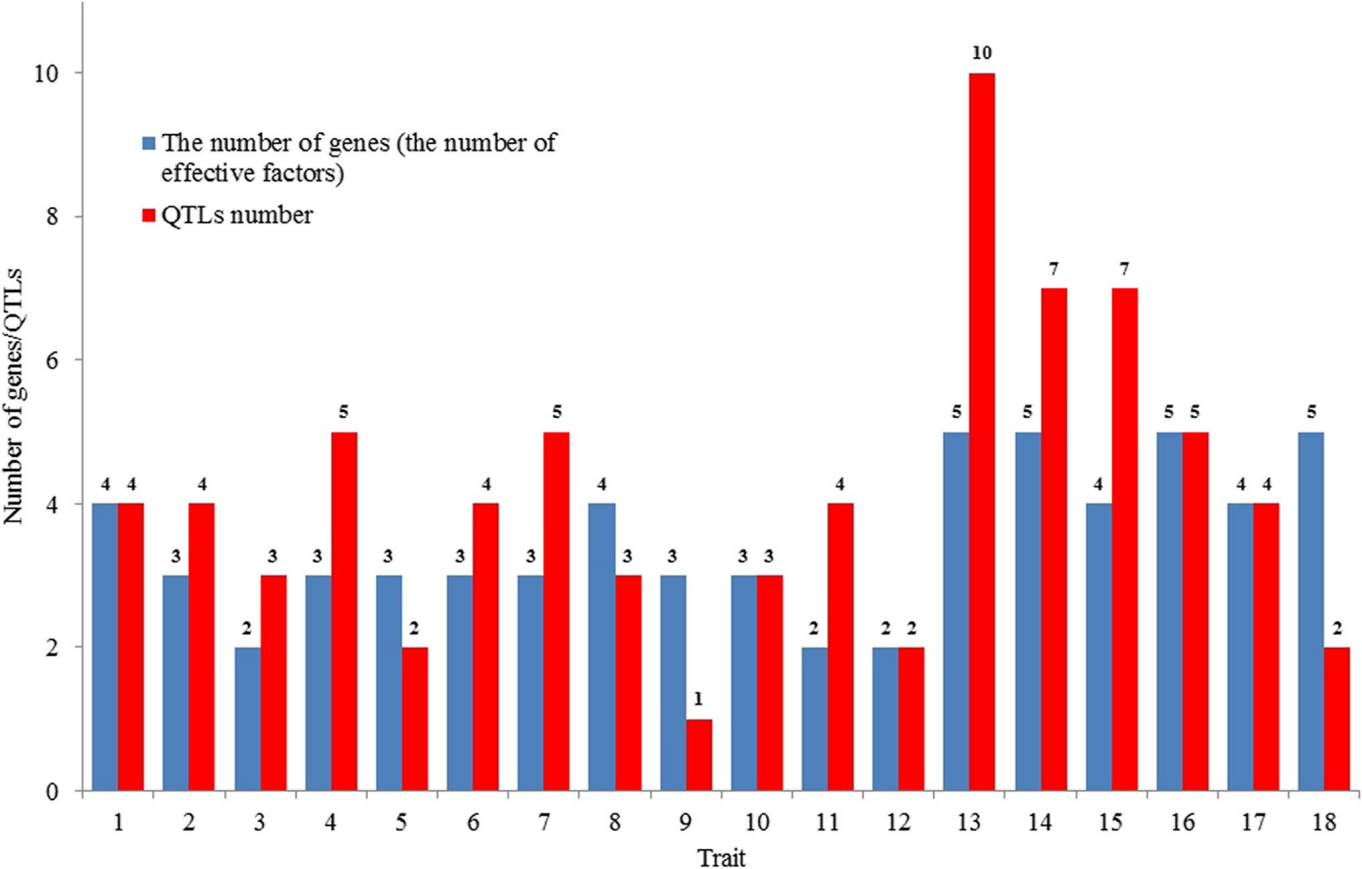
The number of genes (the number of effective factors) estimated on the basis of only phenotypic observations and the number of QTLs for particular traits

Figure 1 shows the number of genes (effective factors) estimated based on only phenotypic observations and the number of QTLs for each trait.

### Estimation based on the phenotype

Phenotypic estimates of the total additive-by-additive-by-additive effect (*aaa*_*p*_) are shown in Table 1. In 13 of 18 cases, the observed *aaa*_*p*_ effect was positive. The effect was negative for traits numbered: 5, 9, 11, 14, and 15. The highest positive *aaa*_*p*_ effect was observed for trait 10 (15.96); the lowest was for trait 9 (− 26.84). This observation came from the same group of isolates (IPO88004, traits 9–12) (Table 1). Twelve of the 18 (66.67% of all cases) *aaa*_*p*_ effects were statistically significant. Eight significant effects were positive, and four were negative (Table 1).

The number of genes (effective factors) varied among traits and groups. The highest was observed for traits 13, 14, 16, and 18 (5 genes); the lowest for traits 3, 11, and 12 (2 genes) (Table 1).

The minimal and maximal line averages were higher for the percentage of necrosis leaf area than for the percentage of leaf area covered by pycnidia. The same is true for the total line average for the mentioned traits. For traits 13–15 (heading data) and 16–18 (height), the differences in means were marginal (Table 1).

### Estimation based on the genotype

The number of genes (the number of effective factors) estimated based on only phenotypic observations and the number of QTLs for each trait are shown in Fig. 1.

The highest number of QTLs can be observed for trait 13 (10, heading data), and the lowest for trait 9 (1, percentage of necrosis leaf area) (Fig. 1).

The number of genes was lower than the number of QTLs in nine cases (traits: 2, 3, 4, 6, 7, 11, 13, 14, and 15), higher in four cases (traits: 5, 8, 9, and 18), and equal in five cases (traits: 1, 10, 12, 16, and 17) (Fig. 1).

#### Unweighted regression

Genotypic estimates of the total additive-by-additive-by-additive effect estimated based on unweighted (*aaa*_*gu*_) and weighted (*aaa*_*gw*_) multiple linear regression are shown in Tables 2 and 3.

The number of significant *aaa*_*gu*_ ranged from 0 (traits: 1–2, 5–6, 9–10, 12, 15, 16–18) to 9 (trait: 4). The total *aaa*_*gu*_ effect ranged from − 14.74 (trait 4) to 15.61 (trait 3) (Table 2).

Compared to phenotypic estimates, the total *aaa*_*gu*_ effects are very different. A positive effect was observed for traits 3 and 8 for both phenotypic and genotypic unweighted observations, while a negative effect was not aligned in any trait. However, the absolute value is higher for the total *aaa*_*gu*_ effect for traits 3, 4, and 7 (Tables 1 and 2).

#### Weighted regression

The number of significant *aaa*_*gw*_ ranged from 0 (traits: 1–2, 5–10, 12, 16–18) to 10 (trait: 13). The cases with 0 significant threes are similar to the results of the unweighted regression, except for traits 7 and 8 (significant only for unweighted) and 15 (significant only for weighted). The total *aaa*_*gw*_ effect ranged from − 23.39 (trait: 4) to 21.65 (trait: 3) (Table 2). The absolute values of the total effect were also larger for the weighted regression than for the unweighted variant for all cases where comparison was possible (Table 2). The number of detected threes by weighted regression was higher in two cases (traits: 13 and 15), lower in four cases (traits: 4, 7, 8, and 14), and the same for the rest (Table 2).

Genotypic estimates of additive-by-additive-by-additive interaction effects for individual QTL × QTL × QTL threes based on unweighted (*aaa*_*gu*_) and weighted (*aaa*_*gw*_) multiple linear regression are shown in Table 3. Forty-nine statistically significant threes were observed. The QTLs most frequently found in threes were as follows: 4,407,404 (9 times), 1,678,083|F|0–32:T > C-32:T > C (7 times), 1,021,903|F|0–14:G > A-14:G > A (7 times), 3,028,296 (7 times), 992,306|F|0–7:A > G-7:A > G (7 times), 3,021,909 (7 times), 1,110,543|F|0–16:C > T-16:C > T (6 times), 2,257,522|F|0–45:C > T-45:C > T (6 times), and 1,226,085 (6 times) (Table 3). Using unweighted regression, the genes most frequently found in threes were as follows: 1,678,083|F|0–32:T > C-32:T > C (7 times), 2,257,522|F|0–45:C > T-45:C > T (6 times), and 1,110,543|F|0–16:C > T-16:C > T (5 times) (Table 3). Using weighted regression, the genes most frequently observed in threes were as follows: 1,678,083|F|0–32:T > C-32:T > C (7 times), 4,407,404 (6 times), 992,306|F|0–7:A > G-7:A > G (6 times), 3,028,296 (6 times), 1,110,543|F|0–16:C > T-16:C > T (5 times), 3,021,909 (5 times), 1,226,085 (5 times), and 2,257,522|F|0–45:C > T-45:C > T (4 times) (Table 3).

For the percentage variance accounted for by the individual QTL × QTL × QTL threes based on unweighted and weighted multiple linear regression, the models are better fit by weighted regressions in all cases (Table 4). The *R*^2^ coefficients for the weighted regressions ranged from 9.6% (trait 4) to 51.8% (trait 4). These values were higher than the coefficients for unweighted regression from 0.8% (trait 3) up to 15.5% (trait 4) (Table 4).

## Discussion

The breeding process aims to obtain genotypes with traits superior to parental forms (Cullis et al. 2020). Decisions on the suitability of breeding material can be influenced by genes with significant additive effects, as well as by the interactions of these genes (epistatic and higher orders) (Bocianowski et al. 2019; Voss-Fels et al. 2019; Ali et al. 2020; Labroo et al. 2021; Raffo et al. 2022). A major challenge in the post-genomic era, especially in estimating QTL effects, QTL-QTL interactions (Yang et al. 2007) and QTL–QTL–QTL interactions (Cyplik et al. 2023), is understanding the genetic architecture of quantitative traits.

Breeding programs using QTLs should consider not only epistatic effects, but also higher-order interactions. To understand the genetic basis of quantitative traits, it is important to determine the contribution of QTL–QTL–QTL triplet interactions. The assumption of the absence of QTL–QTL–QTL triple interaction in genetic QTL mapping models can lead to incorrect estimation (underestimation) of parameters related to QTL effects and their QTL–QTL epistasis interactions (Bocianowski 2013).

The paper presents a numerical comparison of three methods for estimating additive–additive–additive interaction effects. The comparison was carried out on 126 doubled haploid lines (DHLs) of winter wheat obtained from the Mandub (the German cultivar revealed a high level of resistance at the seedling and adult plant stages) × Begra (susceptible cultivar) (Piaskowska et al. 2021). The lines were analyzed for 18 traits, including percentage of necrosis leaf area, percentage of leaf area covered by pycnidia, heading data and height. The present results demonstrated the use of weighted regression to determine the triplets of QTLs and estimate the effects of their QTL–QTL–QTL interaction. Consistent with the best literature, only Cyplik et al. (2023) previously used weighted regression to evaluate QTL-QTL-QTL triple interaction. However, they used a different approach, using weighted regression at all three stages—for QTL selection, epistatic pairs, and QTL–QTL–QTL triples. The consequence of this approach was to obtain different QTL–QTL–QTL triples (in 100% of cases) for both approaches: unweighted regression and weighted regression. The paper uses weighted regression for already selected QTLs. Thirty-one selected QTLs yielded a total of 75 associations for 18 traits. This is a larger number of QTLs than those obtained previously using linkage mapping performed on the same data (Piaskowska et al. 2021). Piaskowska et al. (2021) detected 23 QTLs: 12 QTLs associated with resistance to STB and 11 QTLs associated with plant height or heading date.

In their Monte Carlo simulation study, Cyplik and Bocianowski (2023) considered 84 different experimental situations, comparing estimates of the parameter associated with the triple interaction effects of *aaa* obtained by four methods: a phenotypic method and three genotypic methods (one unweighted and two weighted). One of the weighted regression variants used in the numerical comparisons in the studies presented here proved to be the best method in terms of the closest estimates of the assumed true value of *aaa*, the smallest mean squared errors of the estimates and the largest coefficients of determination characterizing the goodness of the model. The use of weighted regression always yielded higher, in absolute value, *aaa* estimates of gene–gene-gene interaction effects the use of unweighted regression. Compared to phenotypic estimates, the total *aaa*_*gu*_ effects are very different. A positive effect was observed for traits 3 and 8 for both phenotypic and genotypic unweighted observations, and a negative effect was not aligned for any trait. However, the absolute value is higher for the total *aaa*_*gu*_ effect for traits 3, 4 and 7. The coefficients of determination for the models including weights were larger than those for the traditional unweighted regression. For the percentage variance accounted for by the individual QTL × QTL × QTL threes based on unweighted and weighted multiple linear regression, the models are better fitted for weighted regressions in all cases. The coefficients values ranged from 9.6% (trait 4) to 51.8% (trait 4). Values were higher, ranged from 0.8% (trait 3) up to 15.5% (trait 4).

For four of the eighteen (22.22%) traits, the number of QTL was less than three, resulting in the apparent lack of any possibility of QTL–QTL–QTL triples. For seven of the eighteen (38.89%) traits, at least three QTLs were identified, but there were no significant QTL–QTL–QTL triples. More favorable, smaller numbers above could be obtained using lower restrictions than the assumed false positive probability of 0.001. However, it was assumed that highly significant QTLs and their triples were selected (for triples also *p* < 0.001) to make the results as useful as possible for breeding and selection programs, among others.

Thirty-one different QTLs accounted for 38 different QTL–QTL–QTL threes. The most frequently occurring QTLs in threes were: 4407404 (9 times) as well as 1678083|F|0–32:T > C-32:T > C, 1021903|F|0–14:G > A-14:G > A, 992306|F|0–7:A > G-7:A > G, 3021909 and 3028296 (7 times). Twenty-four different QTLs accounted for 24 different significant QTL–QTL–QTL triplets for the unweighted regression. The marker 1678083|F|0–32:T > C-32:T > C was the most frequent (7 times) in the triplets. Twenty-six different QTLs accounted for 25 different significant QTL–QTL–QTL triplets for the weighted regression. The marker 1678083|F|0–32:T > C-32:T > C was the most frequent (7 times) in the triples for the weighted regression. Eleven of 38 (28.95%) QTL–QTL–QTL triplets were significant for the same trait using unweighted and weighted regression. Thirteen (34.21%) of the QTL–QTL–QTL triples were significant using unweighted regression but not using weighted regression. Fourteen of the 38 (36.84%) QTL–QTL–QTL triplets were significant using weighted regression, while they were not significant using traditional unweighted regression. None of the QTL–QTL–QTL triples were significant for more than one of the observed traits.

The selected QTLs were located on nine chromosomes: 1B, 2A, 2B, 2D, 3A, 4A, 4B_2, 7A and 7B. Piaskowska et al. (2021) detected 23 QTLs located on six chromosomes: 1B, 2B, 2D, 4B_2, 5B and 7B. In 19 of 38 cases (50%), all three QTLs forming the QTL–QTL–QTL triplet were located in a single linkage group. In 14 cases (36.84%), only two of the three QTLs forming the triplet were located in one linkage group. In contrast, in five cases (13.16%), all three QTLs forming the QTL–QTL–QTL triplet were located in three different linkage groups. The results obtained are very interesting and require further study. The number of linkage groups containing QTLs forming the QTL–QTL–QTL triplet may be one of the parameters assumed in simulation studies.

## Conclusions

Estimation of higher-order interactions is usually ignored, not because they are unimportant, but because of the high requirement for data. Higher-order interactions are very common and have a huge impact on phenotype. The results show that when using weighted regression on marker observations, the resulting estimates have a higher absolute value than when using unweighted regression. The triple interaction had a significant effect on the expression of the quantitative traits studied. The proposed weighted regression method for estimating the parameter connected with the additive-by-additive-by-additive of gene-by-gene-by-gene interaction effect can bridge the gap between the phenotypic and genotypic methods. The methods presented were a useful statistical tool for QTL characterization and allowed estimation QTL–QTL–QTL interactions. Weighted multiple linear regression, with weights being the inverse of the variances for each line, is a useful statistical tool for estimating the parameter associated with the additive-additive-additive interaction effect (*aaa*).

## Data Availability

The data in this manuscript are available from the corresponding authors upon reasonable request.
